# Whole-Genome Sequencing Reveals Co-Infection with Bovine Viral Diarrhea Virus, Bovine Enterovirus, and Caprine Parainfluenza Virus Type 3 in a Calf from a Cattle Herd in Xizang, China

**DOI:** 10.3390/ani16172805

**Published:** 2026-09-07

**Authors:** Yanan Zhong, Runbo Luo, Kexin Li, Na Li, Chenjun Jiang, Zixuan Li, Jiayan Huang, Kongwang He, Libin Wen, Qi Xiao, Sizhu Suolang

**Affiliations:** 1 Key Laboratory for Prevention and Control of Hydatid Disease in Xizang (Co-Constructed by Ministry and Province), Ministry of Agriculture and Rural Affairs, College of Animal Science, Xizang Agricultural and Animal Husbandry University, Linzhi 860000, China; ynanzhong@126.com (Y.Z.);; 2 Key Laboratory of Veterinary Biological Engineering and Technology, Ministry of Agriculture and Rural Affairs, Jiangsu Academy of Agricultural Sciences, Nanjing 210014, China; 3 Jiangsu Key Laboratory for Food Quality and Safety—State Key Laboratory Cultivation Base of Ministry of Science and Technology, Jiangsu Academy of Agricultural Sciences, Nanjing 210014, China; 4 Jiangsu Co-Innovation Center for Prevention and Control of Important Animal Infections Diseases and Zoonoses, Yangzhou University, Yangzhou 225009, China; 5 Guotai Technology Innovation Center for Veterinary Biological Products (Taizhou), Taizhou 225300, China

**Keywords:** co-infection, bovine viral diarrhea virus, bovine enterovirus, caprine parainfluenza virus type 3, whole-genome sequencing

## Abstract

Although mixed viral infections are increasingly recognized as contributors to bovine diarrhea syndrome, diagnosing such co-infections remains challenging, particularly in high-altitude regions where surveillance is limited. In July 2024, a calf presenting with severe diarrhea and respiratory distress was identified on a cattle farm in Linzhi, Xizang, China. Using unbiased whole-genome sequencing (WGS) of the fecal sample, we assembled near-complete genomes of three distinct RNA viruses: two bovine viral diarrhea virus type 1 (BVDV-1) strains (subtypes 1v and 1q, designated BVDV-1/XZ87 and XZ87), one bovine enterovirus (genotype EV-E, designated BEV/XZ87), and one caprine parainfluenza virus type 3 (CPIV3/XZ87). The CPIV3/XZ87 genome exhibited 99.9% nucleotide identity to the goat-derived GS2017-2 strain from Jiangsu, China, raising the possibility of viral spread through livestock trade. Quantitative real-time PCR (RT-qPCR) confirmed the presence of all three pathogens (Ct values: 24.78 for BEV, 25.98 for CPIV3, and 31.28 for BVDV). This study provides the genomic evidence of a triple co-infection involving BVDV-1, BEV, and CPIV3 in Xizang. It illustrates the potential of WGS for unbiased pathogen detection in complex clinical specimens. The near-complete genomes generated here fill critical gaps in the virological surveillance of this epidemiologically under-sampled high-altitude region.

## Simple Summary

Diarrhea is a major health problem in young cattle, but the causes are often hard to identify—especially in remote, high-altitude regions like Xizang, where disease monitoring is limited. In July 2024, a calf on a farm in Linzhi, Xizang, developed severe diarrhea and breathing difficulties. A sample from the calf was analyzed using advanced genetic technology that can detect any virus present without needing to know what to look for in advance. Three different viruses were found in the same animal: one type of bovine viral diarrhea virus, plus two others—a bovine enterovirus and a virus typically found in goats. The goat-like virus was almost identical to a strain previously found in eastern China, suggesting that animal movements may spread diseases across long distances. These findings demonstrate the value of modern genetic tools for detecting mixed infections that might otherwise be missed. The genetic information obtained in this study can help veterinarians and researchers better understand which viruses are circulating in Xizang and how to protect livestock in this region.

## 1. Introduction

Bovine diarrhea syndrome is a leading cause of morbidity and economic loss in calves worldwide, with a multifactorial etiology involving viral, bacterial, and parasitic agents [1,2,3]. The Qinghai–Tibet Plateau is characterized by low oxygen pressure, intense ultraviolet radiation, and large diurnal temperature variations, which are known to modulate host immune responses and viral fitness. Yet the epidemiological landscape of bovine viral pathogens in this region remains almost entirely unexplored.

Bovine viral diarrhea virus (BVDV), a member of the genus Pestivirus (family Flaviviridae), is one of the most prevalent viral pathogens in cattle worldwide [4]. Its positive-sense RNA genome encodes a single polyprotein that is processed into structural and non-structural proteins [5]. Based on genetic diversity, researchers classify BVDV into three genotypes (BVDV-1, -2, and -3), with BVDV-1 further divided into multiple subgenotypes [6]. BVDV infection can cause a spectrum of clinical manifestations ranging from subclinical to severe diarrhea and reproductive disorders, and its immunosuppressive properties can lead to persistent infection (PI) [7]. Despite its economic importance, the genetic diversity of BVDV circulating in Xizang remains largely uncharacterized.

Bovine enterovirus (BEV), belonging to the species Enterovirus E and F within the family Picornaviridae, is frequently implicated in co-infections and may contribute to disease severity [8]. Given its high prevalence in Chinese herds and synergistic role, BEV warrants inclusion in any comprehensive diagnostic screening for calf diarrhea.

Caprine parainfluenza virus type 3 (CPIV3), a member of the genus Respirovirus within the family Paramyxoviridae, was first identified in goats in eastern China in 2014 and has since been associated with mild to severe respiratory diseases [9]. Recent studies have confirmed its widespread prevalence in Chinese cattle herds [10], highlighting the potential for cross-species transmission between small ruminants and cattle. Notably, CPIV3 shares >70% genome homology with bovine parainfluenza virus type 3 (BPIV3), a significant pathogen involved in the bovine respiratory disease complex (BRDC) [11].

Traditional diagnostic methods, such as PCR and serological assays, are typically designed to detect single pathogens in a targeted manner and may therefore fail to capture complex mixed infections. WGS offers a powerful, unbiased alternative that enables simultaneous identification of multiple pathogens from a single clinical specimen without prior assumptions [12]. In this study, we applied WGS to a fecal sample from a representative calf presenting with concurrent diarrhea and respiratory distress on a large-scale farm in Linzhi, Xizang, aiming to identify potential viral pathogens at the genomic level, recover near-complete viral genomes, and perform phylogenetic characterization to trace their potential origins.

## 2. Materials and Methods

### 2.1. Sample Sources

**Figure 1:**
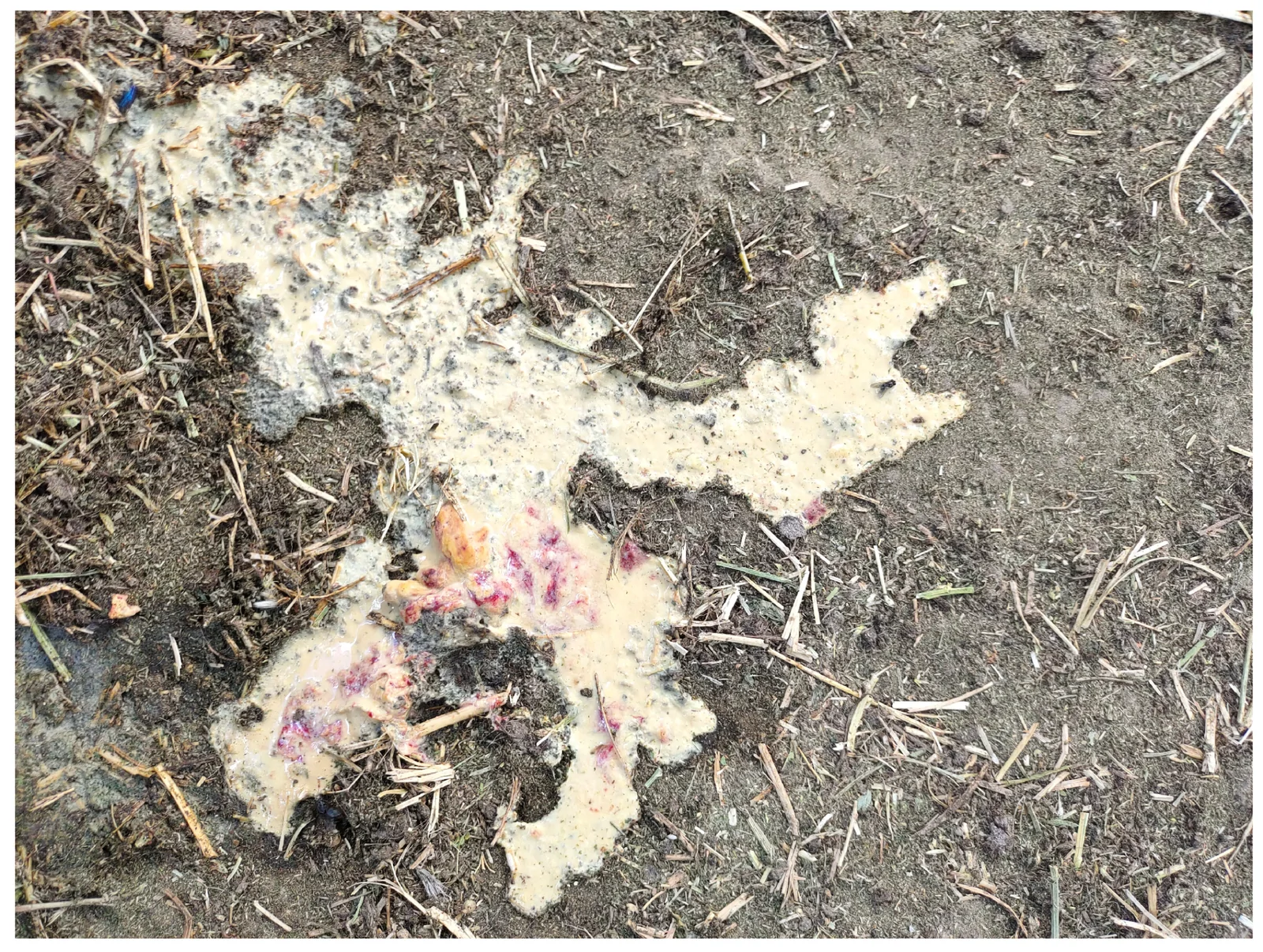
Fecal appearance of the diarrheic calf.

Samples were collected from a large-scale cattle farm in Linzhi, Xizang. In July 2024, calves on the farm exhibited severe diarrhea, lethargy, and concurrent respiratory symptoms, including tachypnea, with clinical manifestations highly suggestive of BVDV infection. Clinical signs persisted despite treatment. Fresh feces were collected from one affected calf (Figure 1), refrigerated, and transported to the laboratory for RNA extraction and viral pathogen detection.

### 2.2. RNA Extraction, Library Construction, and Sequencing

Fresh fecal samples were first homogenized in phosphate-buffered saline (PBS). The homogenate was centrifuged at 3000× *g* for 10 min at 4 °C to remove coarse debris, and the supernatant was collected. To further reduce host-derived contamination, this supernatant was subjected to an additional centrifugation at 12,000× *g* for 10 min at 4 °C. The resulting supernatant was then used for RNA extraction with TRIzol^®^ Reagent (Cat. 15596026, Invitrogen, Carlsbad, CA, USA) following the manufacturer’s instructions. RNA integrity was assessed on an Agilent 2100 Bioanalyzer (Agilent Technologies, Inc., Santa Clara, CA, USA). RNA was fragmented using divalent magnesium cations (Mg^2+^) at 94 °C for 5 min to obtain fragments of approximately 300–500 bp. Fragmented RNA was reverse-transcribed using random hexamers and SuperScript IV Reverse Transcriptase (Cat. 18090010, Invitrogen, Carlsbad, CA, USA) to generate first-strand cDNA. Second-strand cDNA synthesis was performed using the NEBNext^®^ Second Strand Synthesis Module (Cat. E6111L, New England Biolabs, Inc., Ipswich, MA, USA). The double-stranded cDNA was then subjected to end-repair, A-tailing, and adapter ligation using the TruSeq DNA Sample Prep Kit (Cat. FC-121-2001, Illumina, San Diego, CA, USA). Index sequences were added via limited-cycle PCR. Library fragments between 200 and 500 bp were selected using AMPure XP beads (Cat. A63881, Beckman Coulter, Inc., Brea, CA, USA). Cluster generation was carried out on the flow cell using the TruSeq PE Cluster Kit (Cat. PE-401-3001, Illumina, San Diego, CA, USA) with bridge amplification. Finally, paired-end sequencing (2 × 150 bp) was performed on an Illumina NovaSeq 6000 system (Illumina, San Diego, CA, USA).

### 2.3. Bioinformatics Analysis

Raw reads in FASTQ format were processed using fastp v0.20.0 with the following parameters [13]. Adapter sequences were automatically trimmed; the first 30 bp from both ends were removed because FastQC analysis revealed a consistent drop in base quality and biased nucleotide composition (GC skew) in the first 10–15 cycles. Hard-trimming was therefore applied before sliding-window quality trimming, thereby avoiding over-trimming of internal high-quality regions; bases other than A, G, C, T at the 5′ end were removed; a sliding window (size 4, mean quality threshold Q20) was applied to trim low-quality bases; reads containing more than 10% ambiguous bases (N) were discarded; reads shorter than 50 bp after trimming were excluded. Cleaned reads were aligned against the Bos taurus reference genome (ARS-UCD1.2), the SILVA rRNA database v138 (including 16S, 23S, 5S, and ITS sequences), and the NCBI RefSeq bacterial genome collection using BBMap v38.51 with parameters: minid = 0.95, maxindel = 3, k = 13. Reads that did not map to these reference sets were retained as clean reads for de novo assembly. The clean reads were assembled de novo using SPAdes v3.14.1 in metagenomic mode (--meta) with k-mer sizes of 21, 33, 55, 77, 99, and 127 [14]. Contigs shorter than 500 bp were discarded.

Assembly completeness was assessed by mapping the clean reads back to the assembled contigs using Bowtie2 v2.4.5 [15]. Assembled contigs were compared against the NCBI virus-NT database using BLASTn v2.10.0 with an e-value threshold of 1×10^−5^ [16,17]. Contigs showing >90% coverage and >85% nucleotide identity to known viral genomes were selected as candidate viral sequences.

For BVDV, which yielded two distinct subgenotypes (1v and 1q) from the same sample, an additional subgenotype deconvolution step was performed. All BVDV-like contigs were mapped against reference sequences of BVDV-1v (ON901785) and BVDV-1q (JN400273) using Bowtie2. Reads that mapped preferentially (≥95% identity, ≥80% read length coverage) to either reference were assigned to that subgenotype, followed by independent SPAdes assemblies for each subset. For all candidate viral genomes, read depth and coverage were calculated using SAMtools v1.15.1 after read mapping with Bowtie2 [15,18], using a sliding window of 100 bp.

Coding sequence (CDS) prediction was performed using Prokka (v1.14.5), which integrates external tools including Prodigal (v0.9) and RNAmmer (v1.2) to predict gene models and other genomic features within contigs. The identified CDSs were then subjected to BLASTp searches against the NCBI non-redundant (NR) database and the UniProt database (https://www.uniprot.org). It should be noted that, as 5′ and 3′ RACE were not performed, the terminal sequences were inferred by reference-guided alignment; consequently, all assembled genomes are designated as near-complete rather than full-length.

### 2.4. Sequence Alignment and Phylogenetic Analysis

Reference sequences of the target viruses, including BVDV, BEV, and CPIV3 (including BPIV3 and ovine parainfluenza virus type 3 (OPIV3) reference strains), were downloaded from the GenBank database (Table 1, Table 2 and Table 3). Functional region annotation of the assembled viral genomes was performed using Geneious Prime 2025.0.2 software with reference sequences as templates to determine the start and end positions of each gene, generating annotated genome sequence files.

Whole-genome nucleotide sequence alignment was performed using the Clustal W algorithm in the MegAlign Pro module of DNAStar Lasergene 17.1. Nucleotide similarities were then calculated for the complete genome and individual genes. Nucleotide similarity was also calculated for each individual gene. A homology heatmap was generated using the ImageGP online tool (https://www.bic.ac.cn/ImageGP, accessed on 27 January 2026) to visually display the homology differences between the strains in this study and the reference strains [19].

Multiple sequence alignment of the complete virus genomes was performed using MEGA 12.0 software with the Clustal W algorithm. The optimal fitting model was selected using the ModelTest module, and a maximum likelihood (ML) phylogenetic tree was constructed. The reliability of the tree nodes was evaluated by bootstrap analysis with 1000 replicates [20].

### 2.5. RT-qPCR Detection

To validate the viral sequences identified by WGS, a probe-based RT-qPCR assay was performed on the same fecal RNA sample. The primers and probes for BVDV were designed according to the Chinese National Standard GB/T 18637-2018 [21], with the forward primer 5′-CCGCGAMGGCCGAAAAGA-3′, reverse primer 5′-TGACGACTNCCCTGTACTCAG-3′, and probe FAM-CCATGCCCTTAGTAGGACTAGCA-BHQ1. The BEV primers (forward: 5′-ATGCTGCTAATCCCAACCTC-3′; reverse: 5′-GAGTACCGAAAGTAGTCTGTTC-3′) and probe (VIC-ACAATCCAGTGTTGCTACGTCGTAACG-MGB) were used as previously described [22]. The CPIV3 primers (forward: 5′-GCTTGGCTTCTTTGAAATGG-3′; reverse: 5′-GCCTGCAGAAGTTCCTTGTC-3′) and probe (FAM-CAATCGGACTAGCCAAGTATGGTGGGA-BHQ1) were designed according to the Chinese National Standard GB/T 44613-2024 [23].

cDNA synthesis was performed separately using HiScript II 1st Strand cDNA Synthesis Kit (+gDNA wiper) (Cat. R212-02, Nanjing Vazyme Biotech Co., Ltd., Nanjing, China). Briefly, genomic DNA was removed by incubating RNA with 4 × gDNA wiper Mix at 42 °C for 2 min in a 16 μL reaction. First-strand cDNA was then synthesized by adding 2 μL of 10 × RT Mix, 2 μL of HiScript II Enzyme Mix, and 1 μL of primer (Oligo (dT)_23_VN/Random hexamers/gene-specific primers) to the above mixture, bringing the final volume to 20 μL. The reverse transcription reaction was carried out at 50 °C for 45 min, and 85 °C for 5 s to inactivate the enzyme. qPCR was performed using the AceQ qPCR Probe Master Mix (Vazyme, Q112-02) on a CFX96™ Real-Time PCR Detection System (Bio-Rad Laboratories, Inc., Hercules, CA, USA). The thermal cycling protocol was: 95 °C for 5 min, followed by 40 cycles of 95 °C for 10 s and 60 °C for 30 s. As no positive plasmids were available, absolute viral loads (copy numbers) were not determined in this study. No internal reference gene was included because the RT-qPCR assay was used solely for qualitative validation of the viruses identified by WGS, not for inter-sample normalization or absolute quantification. Accordingly, Ct values are reported only as detection indicators, and direct quantitative comparisons across different viral targets are not intended. A non-template control (NTC) was included as negative control in each run. As this study was based on a single clinical sample, the RT-qPCR results were used solely for validation of the WGS findings and not for quantitative comparison of viral loads between different pathogens.

## 3. Results

### 3.1. Sequencing Data and Assembly

Illumina NovaSeq 6000 sequencing generated a total of 9,740,624 reads, corresponding to 1,461,093,600 bp. Assessment of the raw data showed that the quality scores of all base positions were within the high-quality range, with Q20 and Q30 ratios of 97.91% and 91.96%, respectively, indicating high accuracy of the sequencing data. The per-base sequence content distribution revealed fluctuations in the base ratios within the first 5 bp of the reads, while the base composition stabilized after the first 10 bp, which is characteristic of high-throughput sequencing data.

After filtering rRNA sequences, host genomic sequences, and bacterial sequences using the BBMap software suite, 9,588,276 high-quality clean reads were obtained, with a total base length of 1,390,920,072 bp. The Q20 and Q30 ratios further increased to 98.67% and 93.36%, respectively. The read length was slightly shortened, but the data quality met the requirements for subsequent analyses. De novo assembly of the clean reads, followed by completeness assessment and taxonomic annotation, generated near-complete genomes corresponding to four viral sequences of BVDV-1, BEV, and CPIV3. The assembly statistics for the three viral genomes are provided in Table 4.

### 3.2. Virus Identification

**Figure 2:**
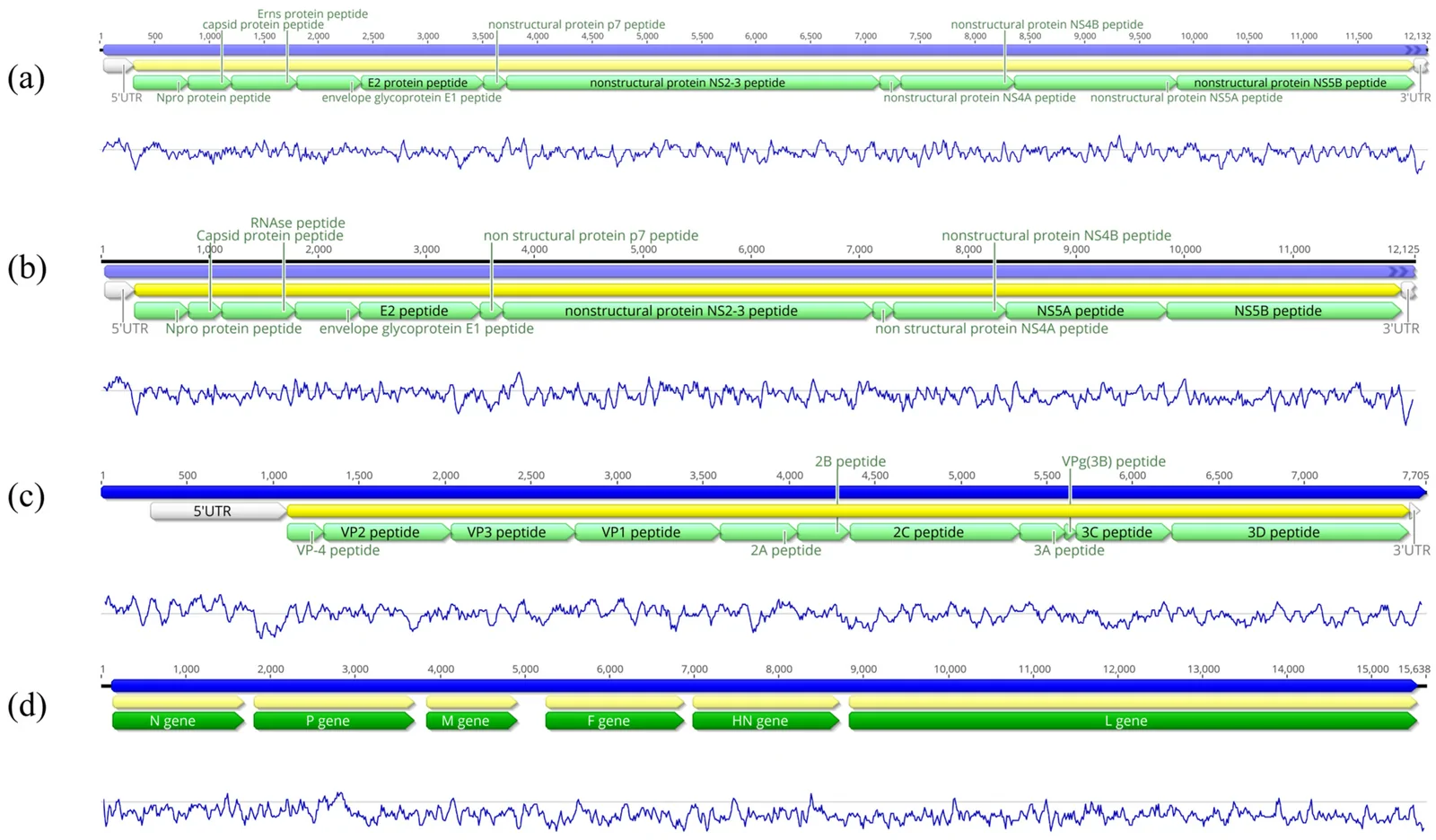
Whole-genome gene annotation of the assembled viruses. (**a**) BVDV-1/XZ87; (**b**) XZ87; (**c**) BEV/XZ87; (**d**) CPIV3/XZ87.

Two BVDV-1 strains were identified: one designated BVDV-1/XZ87 with a genome length of 12,132 bp (GenBank accession no. PX684447), and the other designated XZ87 with a genome length of 12,125 bp (GenBank accession no. PX556467). The genomes contained the canonical ORF, 5′-UTR, and 3′-UTR characteristic of the genus Pestivirus within the family Flaviviridae, consistent with the genomic structure of BVDV-1 (Figure 2a,b).

A near-complete BEV genome was obtained, designated BEV/XZ87, with a length of 7705 bp (GenBank accession no. PX684448). Its genomic structure comprised the P1, P2, and P3 coding regions typical of the genus Enterovirus, with P1 encoding the viral capsid proteins (VP1, VP2, VP3, VP4) and P2 and P3 encoding non-structural proteins, consistent with the known BEV genome organization (Figure 2c).

A near-complete CPIV3 genome was also obtained, designated CPIV3/XZ87, with a length of 15,638 bp (GenBank accession no. PX684449). The genome harbors six structural genes encoding the N, P, M, F, HN, and L proteins, consistent with the canonical CPIV3 genomic structure (Figure 2d).

### 3.3. Phylogenetic Analysis

#### 3.3.1. Genetic Evolutionary Analysis of BVDV

Phylogenetic analysis of the near-complete BVDV-1 genomes was conducted using the ML method (Figure 2a). The BVDV-1/XZ87 strain formed a terminal clade with high bootstrap support alongside the BVDV-1v subtype strain BVDV-1/Bovine/CHN/HB-03/2017, isolated from Hubei Province, China (GenBank accession no. ON901785.1). Additionally, the XZ87 strain showed a close phylogenetic relationship with the BVDV-1q subtype strain SD0803, isolated from Shandong Province, China (GenBank accession no. JN400273.1), as reflected by their tight clustering.

**Figure 3:**
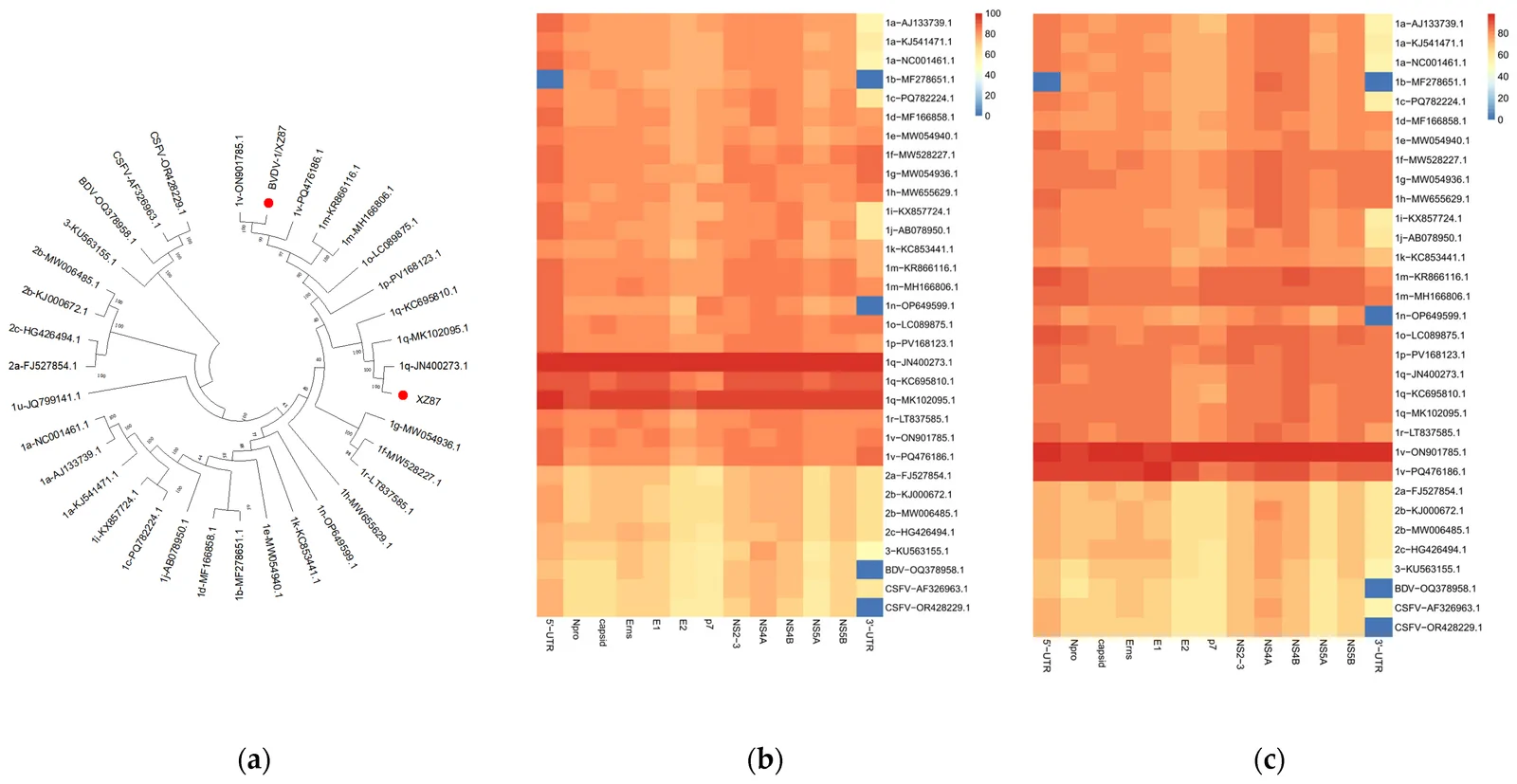
Phylogenetic tree and nucleotide homology of BVDV. (**a**) BVDV phylogenetic tree; (**b**) nucleotide homology of the XZ87 strain; (**c**) nucleotide homology of the BVDV-1/XZ87 strain. Note: 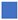 Indicates missing data; 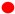 Represent the XZ87 and BVDV-1/XZ87 strains, whose complete genomes were sequenced in this study.

Homology analysis revealed that BVDV-1/XZ87 shared the highest whole-genome nucleotide identity (95.6%) with the BVDV-1/Bovine/CHN/HB-03/2017 strain, and high nucleotide identities were also observed between each gene of BVDV-1/XZ87 and its counterpart in this reference strain (Figure 3b). Similarly, the XZ87 strain exhibited a high whole-genome nucleotide identity of 98.8% with the SD0803 strain, with similarly high levels observed across all individual genes (Figure 3c). In contrast, the E2 gene—a major antigenic gene of BVDV—showed 77.2% nucleotide identity and 79.1% amino acid identity between BVDV-1/XZ87 and the BVDV-1q strain, suggesting potential antigenic differences between the two BVDV-1 subtypes. Phylogenetic and nucleotide homology analyses collectively confirmed that BVDV-1/XZ87 belongs to subtype BVDV-1v, whereas the comparator strain is classified as BVDV-1q.

#### 3.3.2. Genetic Evolutionary Analysis of BEV

**Figure 4:**
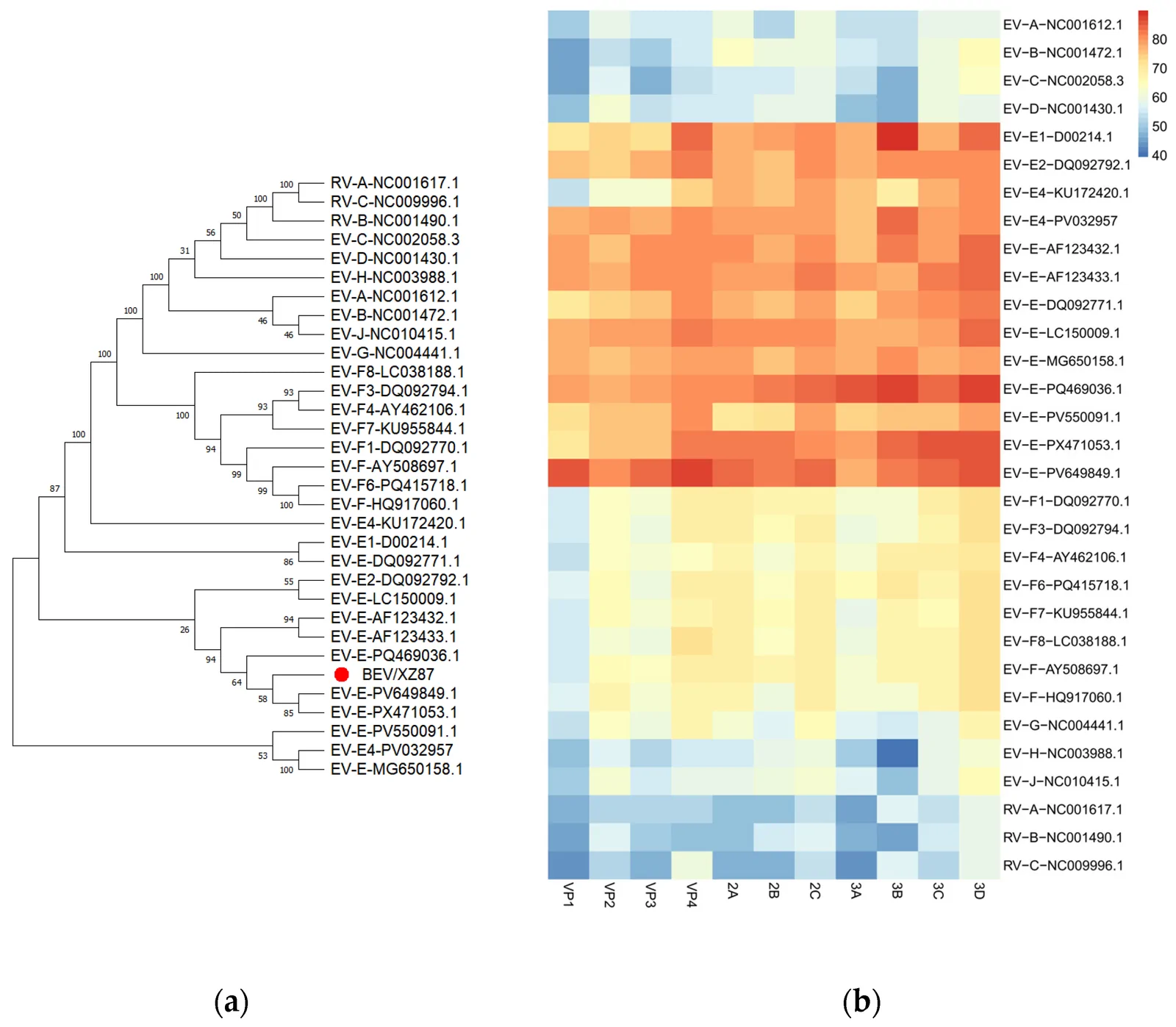
Phylogenetic tree and nucleotide homology of BEV. (**a**) BEV/XZ87 phylogenetic tree; (**b**) nucleotide homology of the BEV/XZ87 strain. 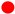 Represent the BEV/XZ87 strains, whose complete genomes were sequenced in this study.

Phylogenetic analysis of BEV/XZ87 (Figure 4a) showed that it clustered closely with the EV-E genotype strain BEVGX06, isolated from Guangxi, China (GenBank accession no. PV649849.1), confirming that BEV/XZ87 belongs to the EV-E genotype. Whole-genome homology alignment revealed that BEV/XZ87 shared 84.5% nucleotide identity with BEVGX06, with nucleotide similarities for each gene fragment (P1, P2, and P3 regions) all below 90%, and even lower homology to reference strains of other genotypes (Figure 4b).

#### 3.3.3. Genetic Evolutionary Analysis of CPIV3

**Figure 5:**
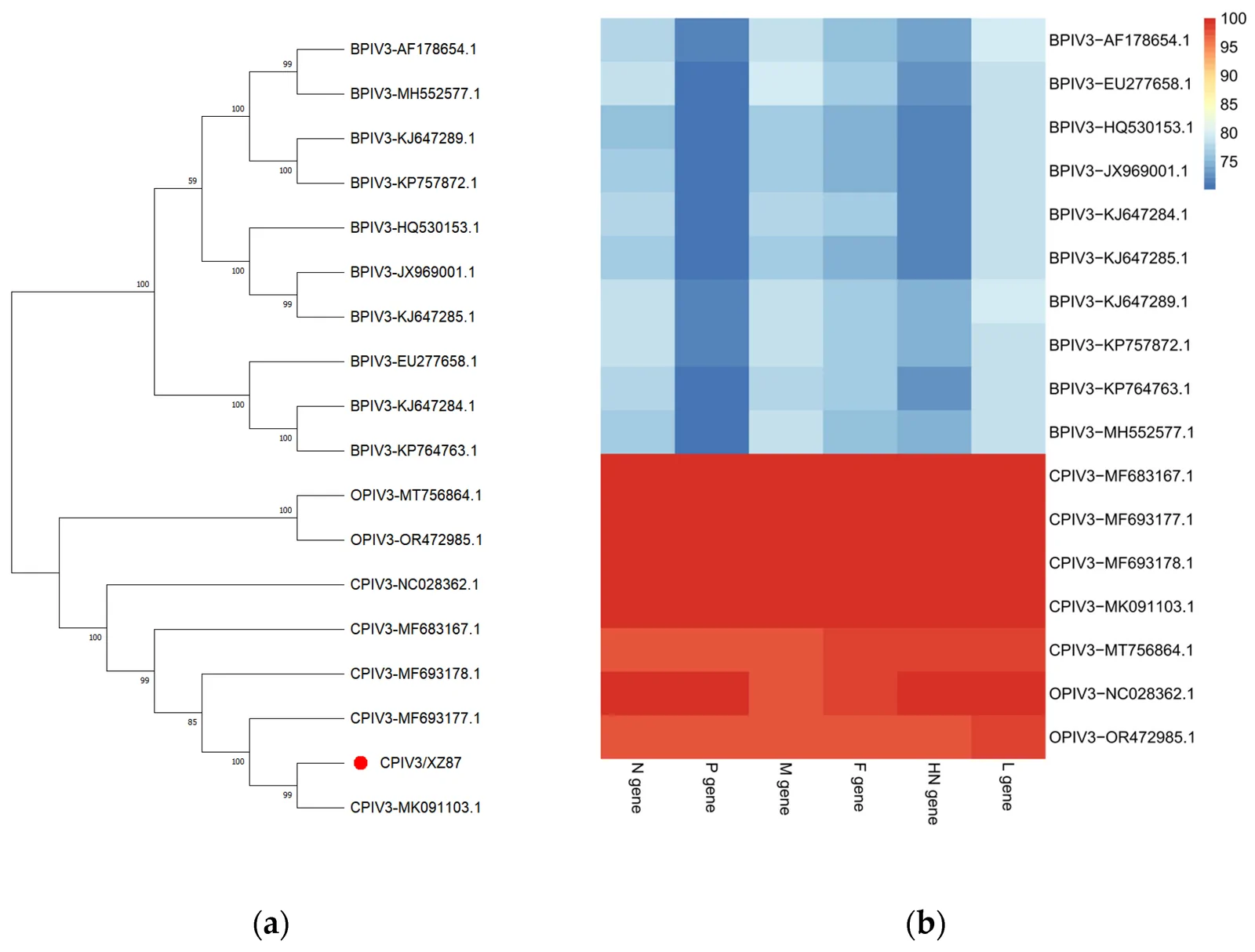
Phylogenetic tree and nucleotide homology of CPIV3. (**a**) CPIV3/XZ87 phylogenetic tree; (**b**) nucleotide homology of the CPIV3/XZ87 strain. 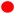 Represent the CPIV3/XZ87 strains, whose complete genomes were sequenced in this study.

Phylogenetic analysis of CPIV3/XZ87 (Figure 5a) showed that it clustered completely with the GS2017-2 strain isolated from Jiangsu, China (GenBank accession no. MK091103.1), with strong bootstrap support (>90%), indicating a close evolutionary relationship. Whole-genome homology analysis revealed that CPIV3/XZ87 shared 99.9% nucleotide identity with GS2017-2, with nucleotide identities for each structural gene (N, P, M, F, HN, L) ranging from 99.8% to 100%, and >70% identity to all components of OPIV3 and BPIV3 (Figure 5b). These results confirm the high sequence conservation of core functional genes between the two strains and provide valuable evidence for the epidemiological traceability of CPIV3/XZ87.

### 3.4. RT-qPCR Validation of Viral Genomes

**Figure 6:**
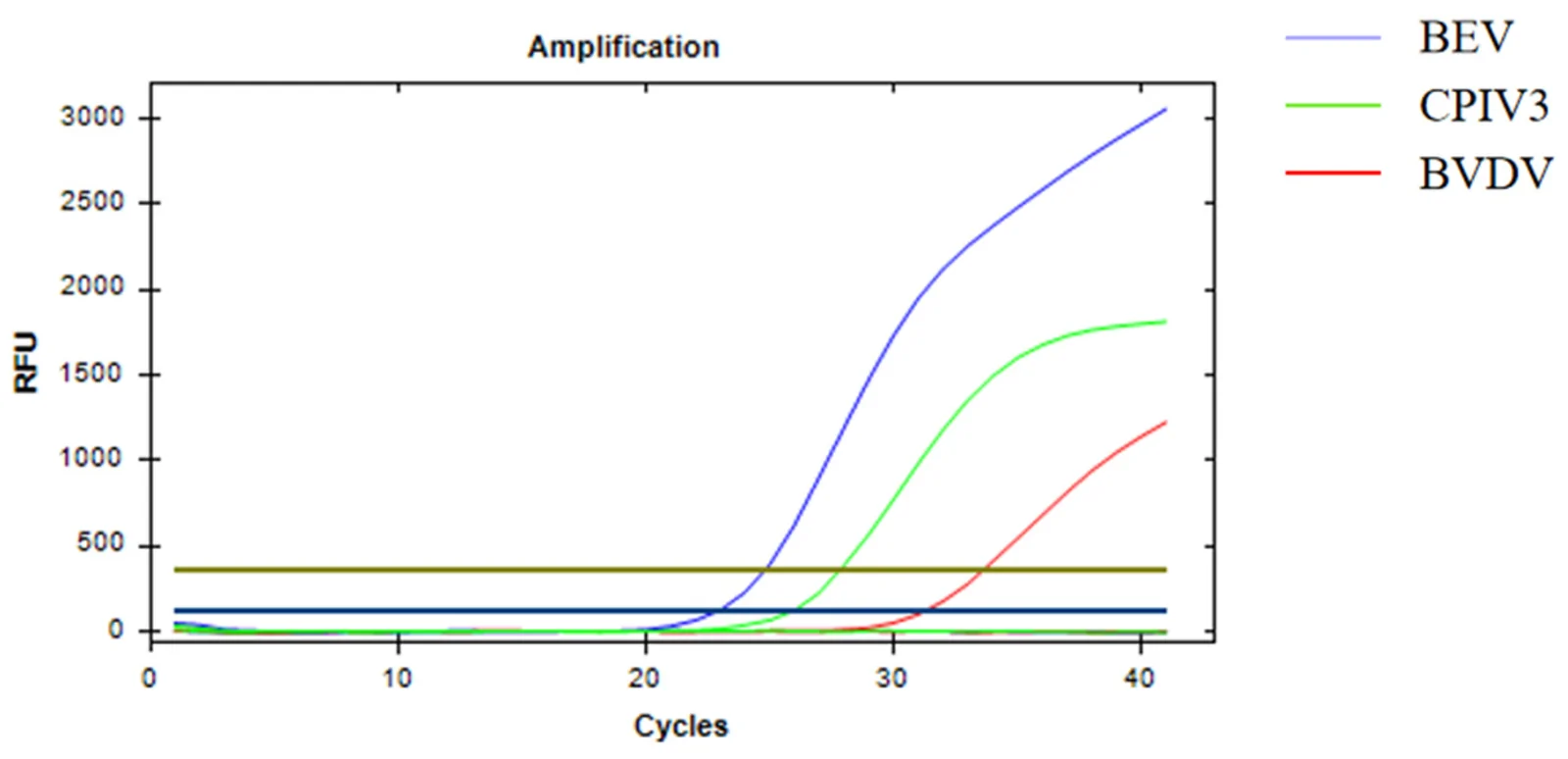
RT-qPCR amplification plots for the detection of BEV, BVDV, and CPIV3 in the fecal sample. The blue horizontal line represents the baseline/threshold for the FAM channel, and the yellow horizontal line represents the baseline/threshold for the VIC channel.

The probe-based qPCR assay successfully detected all three targeted viruses in the fecal sample, with no amplification observed in the NTC (Figure 6). The Ct values for BEV/XZ87, CPIV3/XZ87, and BVDV were 24.78, 25.98 and 31.28, respectively. Given that the RT-qPCR assay targets the conserved 5′-UTR region, the Ct value of 31.28 represents the combined viral load of both BVDV-1 subgenotypes (1v and 1q) present in the sample. The higher Ct value for BVDV-1 relative to those for BEV and CPIV3 suggests a relatively lower abundance of BVDV-1 RNA in this fecal sample.

## 4. Discussion

Bovine diarrhea syndrome is a leading cause of morbidity and mortality in calves, with pathogenesis dependent on the interplay between pathogens, environmental factors, and host susceptibility. Although bovine diarrhea syndrome has a multifactorial etiology, RNA viruses are increasingly recognized as primary etiological agents, especially as research into their epidemic potential and evolutionary dynamics advances [24]. However, the specific viral pathogens circulating in high-altitude regions such as Xizang remain largely unexplored. In this study, we employed unbiased WGS to identify a triple co-infection with BVDV-1, BEV, and CPIV3 in a diarrheic calf from a cattle farm in Linzhi, Xizang. The recovery of near-complete genomes for three distinct viral pathogens from a single fecal sample underscores the power of metagenomic WGS as a frontline tool for unbiased pathogen surveillance, particularly in under-sampled high-altitude regions where the epidemiological landscape remains poorly defined.

CPIV3/XZ87 from the Tibetan calf and the goat-derived GS2017-2 strain from Jiangsu share 99.9% genome identity, suggesting a very recent common ancestor and possible introduction via livestock trade between Jiangsu and Xizang. This is epidemiologically plausible, as increasing animal movement between the Qinghai–Tibet Plateau and low-altitude provinces may facilitate viral spread among ruminants. Notably, CPIV3 has been isolated from cattle herds in China, with a positive rate of 34.21% at the individual level and 70.59% at the farm level, and its high genomic similarity to goat-derived strains suggests transboundary transmission capacity among ruminants [10]. CPIV3 shares over 70% nucleotide identity with BPIV3, a well-established bovine respiratory pathogen, and variations in the HN protein may further expand its host range [10]. While these observations suggest that CPIV3 may pose a prevalence risk, systematic surveillance—including seroprevalence surveys and geographic mapping—is needed to assess its threat to bovine respiratory health. The detection of CPIV3 in a fecal sample is noteworthy, as CPIV3 is primarily a respiratory pathogen. Fecal shedding could result from swallowed respiratory secretions or, alternatively, reflect enteric replication. Gastrointestinal shedding of parainfluenza viruses has been documented in humans and ruminants [25], and BPIV3 RNA has been detected in feces of experimentally infected calves [26]. Although our cross-sectional data cannot distinguish these possibilities, the low Ct value supports active replication rather than passive contamination, highlighting the need for future studies on the potential enteric tropism or fecal–oral transmission of CPIV3 in cattle.

The concurrent detection of two distinct BVDV-1 subgenotypes (1v and 1q) in a single animal highlights the remarkable genetic diversity of BVDV circulating in Xizang. The unique high-altitude ecology of the Tibetan Plateau may promote the dynamic evolution of viral quasispecies, potentially giving rise to divergent subgenotypes within the same host population. This diversity raises concerns about potential antigenic differences between subgenotypes: studies have demonstrated that the NS4B gene yields subgenotyping results most consistent with whole-genome phylogeny, making it a reliable target for subtype identification in BVDV-1 [27]; however, amino acid variations in these genes may affect neutralizing epitopes, potentially compromising the protective efficacy of conventional vaccines [27]. Although commercial vaccines based on subtype 1a may offer partial cross-protection, the antigenic divergence observed in the E2 gene between subtypes 1v and 1q warrants further evaluation of vaccine efficacy against locally circulating strains [28]. Even in vaccinated herds, outbreaks of persistent infections with subgenotypes 1b and 1d have been reported [29], underscoring the need to combine subgenotype surveillance with biosecurity measures.

The concurrent presence of BVDV-1, BEV, and CPIV3 in this calf, which presented with both severe diarrhea and respiratory distress, is generally consistent with the known tissue tropisms of these viruses—BEV as an enteric pathogen and CPIV3 as a respiratory pathogen. Given the systemic immunosuppression induced by BVDV, it is plausible that this co-infection may have contributed to the severity and combined nature of the clinical signs observed in this case. However, owing to the single-animal nature of this study, whether these viruses acted synergistically or independently cannot be determined; the observed association should therefore be interpreted as hypothesis-generating rather than conclusive, and requires validation in larger-scale studies. BVDV infection can create a permissive environment for secondary pathogens by inducing immunosuppression [30]. Mechanistically, BVDV has evolved strategies to evade host innate immunity, notably by impairing type I interferon (IFN) responses through its non-structural proteins [31]. This IFN antagonism may compromise host defenses against concurrently infecting respiratory and enteric viruses, thereby potentially contributing to the dual clinical presentation of diarrhea and respiratory distress observed in this calf. However, this mechanistic hypothesis remains speculative and requires experimental validation using in vitro co-infection models or longitudinal in vivo studies.

Several limitations should be acknowledged. First, the clinical presentation could also involve undetected pathogens, host factors, or environmental stressors. Second, the lack of absolute quantification standards and an internal reference gene in our RT-qPCR assay means the Ct values are semi-quantitative at best and should not be interpreted as viral load rankings. Third, our bioinformatic deconvolution of two BVDV subgenotypes relied on reference-guided read separation, which may not fully resolve the complexity of viral quasispecies; long-read sequencing would be needed for definitive resolution. Fourth, we did not perform 5′/3′ RACE, so the terminal sequences were inferred from reference alignment, and the genomes are therefore designated as “near-complete”. Finally, the generalisability of our findings to other herds or regions is limited, and larger surveillance studies are required. Despite these limitations, our findings provide essential genomic reference data for this epidemiologically critical region and demonstrate the utility of WGS for the precise diagnosis of complex, multi-pathogen infections in livestock.

## 5. Conclusions

This study presents the first genomic evidence of a triple co-infection (BVDV-1, BEV, and CPIV3) in a calf from Xizang, illustrating the potential of unbiased WGS for pathogen discovery in under-sampled regions. The near-identity of the CPIV3 genome with a Jiangsu strain raises a speculative but epidemiologically plausible concern that warrants further investigation into livestock trade routes. These findings provide useful genomic reference data for future surveillance in the region and support the integration of WGS-based approaches into routine diagnostic workflows for high-altitude cattle herds.

## Figures and Tables

**Table 1:** Reference strains of BVDV used in this study.

Accession No	Strain Name	Subgenotype	Isolation Location
AJ133739.1	NCP-NADL	1a	USA
NC001461.1	NADL	1a	USA
KJ541471.1	GS5	1a	Lanzhou, Gansu, China
MF278651.1	XZ01	1b	Xizang, China
PQ782224.1	BVDV-1c-IM-2018	1c	Harbin, Heilongjiang, China
MF166858.1	SWU-DJ2	1d	Tibetan Plateau
MW054940.1	MA/101/05	1e	Italy
MW528227.1	2018BVD01695	1f	Germany
MW054936.1	UM/111/06	1g	Italy
MW655629.1	R3572-90	1h	Switzerland
KX857724.1	ACM/BR/2016	1i	Brazil
AB078950.1	KS86-1ncp	1j	Japan
KC853441.1	SuwaCp	1k	Switzerland
KR866116.1	SD-15	1m	Changchun, Jilin, China
MH166806.1	XC	1m	Xichang, Sichuan, China
OP649599.1	19Q060	1n	South Korea
LC089875.1	IS26/01ncp	1o	Japan
PV168123.1	BVDV-1p-2020	1p	Harbin, Heilongjiang, China
KC695810.1	camel-6	1q	Lanzhou, Gansu, China
MK102095.1	20170226	1q	Shihezi, Xinjiang, China
JN400273.1	SD0803	1q	Shandong, China
LT837585.1	Monopartite	1r	Italy
ON901785.1	BVDV-1/Bovine/CHN/HB-03/2017	1v	Hubei, China
PQ476186.1	NX2019/02	1v	Ningxia, China
FJ527854.1	XJ-04	2a	Yangzhou, Jiangsu, China
KJ000672.1	SD1301	2b	Beijing, China
MW006485.1	HEN01	2b	Changchun, Jilin, China
HG426494.1	SH2210-23	2c	Germany
KU563155.1	HN1507	3	Nanyang, Henan, China
OQ378958.1	HuLB14	BDV	Changchun, Jilin, China
OR428229.1	2639/2014	CSFV	India
AF326963.1	Eystrup	CSFV	Germany

**Table 2:** Reference strains of BEV used in this study.

Accession No	Strain Name	Genotype	Isolation Location
AF123432.1	K2577	EV-E	Australia
AF123433.1	SL305	EV-E	Australia
PQ469036.1	XJ-FHT	EV-E	Wulumuqi, Xinjiang, China
PV550091.1	BEVGX20	EV-E	Nanning, Guangxi, China
PX471053.1	BEV-BL311	EV-E	Urumqi, Xinjiang, China
LC150009.1	BEV IS1/Bos taurus/JPN/1990	EV-E	Japan
MG650158.1	BJ101	EV-E	Beijing, China
DQ092771.1	Vir 404/03	EV-E	Germany
PV649849.1	BEVGX06	EV-E	Nanning, Guangxi, China
D00214.1	VG-5-27	EV-E1	United Kingdom
DQ092792.1	PS 42; ATCC VR-758	EV-E2	USA
KU172420.1	MexKSU/5	EV-E4	USA
PV032957.1	NM2407	EV-E4	Hohhot, Inner Mongolia, China
HQ917060.1	BHM26	EV-F	Harbin, Heilongjiang, China
AY508697.1	3A	EV-F	USA
DQ092770.1	BEV-261	EV-F1	Germany
DQ092794.1	PS87/Belfast; ATCC VR-774	EV-F3	USA
AY462106.1	W1	EV-F4	New Zealand
PQ415718.1	T11f/UK/1960	EV-F6	United Kingdom
KU955844.1	SWUN-AB001	EV-F7	Chengdu, Sichuan
LC038188.1	AN12	EV-F8	Japan
NC001612.1	G-10	EV-A	USA
NC001472.1	Coxsackievirus B1	EV-B	USA
NC002058.3	Human poliovirus 1 Mahoney	EV-C	USA
NC001430.1	Enterovirus 70	EV-D	USA
NC004441.1	UKG/410/73	EV-G	USA
NC003988.1	A-2 plaque virus	EV-H	USA
NC010415.1	1631	EV-J	USA
NC001617.1	HRV89	RV-A	USA
NC001490.1	Human rhinovirus B14	RV-B	USA
NC009996.1	024	RV-C	Hong Kong, China

**Table 3:** Reference strains of CPIV3 used in this study.

Accession No	Strain Name	Genotype	Isolation Location
MK091103.1	GS2017-2	CPIV3	Nanjing, Jiangsu, China
MF693177.1	AHQJ2015-1	CPIV3	Nanjing, Jiangsu, China
MF693178.1	JSHA2016	CPIV3	Nanjing, Jiangsu, China
MF683167.1	JSHA2014-1	CPIV3	Nanjing, Jiangsu, China
NC028362.1	JS2013	CPIV3	Nanjing, Jiangsu, China
OR472985.1	TJ2022	OPIV3	Tianjin, China
MT756864.1	TX01	OPIV3	Huhhot, Inner Mongolia, China
JX969001.1	12Q061	BPIV3	South Korea
KJ647285.1	TVMDL16	BPIV3	USA
HQ530153.1	SD0835	BPIV3	Harbin, Heilongjiang, China
KP764763.1	TtPIV-1	BPIV3	USA
KJ647284.1	TVMDL15	BPIV3	USA
EU277658.1	Q5592	BPIV3	Australia
AF178654.1	Kansas/15626/84	BPIV3	USA
KP757872.1	3/Egypt/2014	BPIV3	Egypt
KJ647289.1	TVMDL60	BPIV3	USA
MH552577.1	BPIV3 BJ	BPIV3	Beijing, China

**Table 4:** Sequencing Data and Assembly.

Genome	Clean Reads	Avg Depth	Median	Coverage%	Cov 10× %
BEV/XZ87	4,794,138	4274.58	4304	100	100
CPIV3/XZ87		1072.82	969	100	99.99
BVDV-1/XZ87		35.35	36	100	99.31
XZ87		50.48	50	100	99.94

## Data Availability

The genome sequence data generated and analyzed in this study have been deposited in the National Center for Biotechnology Information (NCBI) GenBank database under the following accession numbers, and are publicly accessible without restrictions. Bovine Viral Diarrhea Virus (BVDV): Accession No. PX684447 (https://www.ncbi.nlm.nih.gov/nuccore/PX684447.1/, accessed on 25 February 2026) and Accession No. PX556467 (https://www.ncbi.nlm.nih.gov/nuccore/PX556467.1/, accessed on 23 January 2026); Bovine Enterovirus (BEV): Accession No. PX684448 (https://www.ncbi.nlm.nih.gov/nuccore/PX684448.1/, accessed on 25 February 2026); Caprine Parainfluenza Virus Type 3 (CPIV3): Accession No. PX684449 (https://www.ncbi.nlm.nih.gov/nuccore/PX684449.1/, accessed on 25 February 2026). All data are available to the scientific community in accordance with FAIR (Findable, Accessible, Interoperable, Reusable) data principles to ensure full transparency and reproducibility of the results reported herein.

## Abbreviations

The following abbreviations are used in this manuscript:

BVDV	Bovine viral diarrhea virus
BEV	Bovine enterovirus
CPIV3	Caprine parainfluenza virus type 3
WGS	Whole-genome sequencing
RT-qPCR	Reverse transcription quantitative real-time PCR
BPIV3	Bovine parainfluenza virus type 3
ORF	Open reading frame
ML	Maximum likelihood
EV	Enterovirus
RV	Rhinovirus
